# The Influences of Community-Enriched Environment on the Cognitive Trajectories of Elderly People

**DOI:** 10.3390/ijerph18168866

**Published:** 2021-08-23

**Authors:** Shuyang Yu, Meng Wei

**Affiliations:** 1 School of City Management, Beijing Open University, Beijing 100081, China; ysyhappy1018@163.com; 2 National Institute of Social Development, Chinese Academy of Social Sciences, Beijing 100732, China

**Keywords:** community, the elderly, enriched environment, cognitive trajectory

## Abstract

To examine the influences of community-enriched environment on the cognitive trajectories of the elderly in China, using panel data of 10,057, 3994, 2387, and 1749 older persons aged 65–104 years of the 2005, 2008, 2011, and 2014 waves from the Chinese Longitudinal Health Longevity Survey (CLHLS) and a growth curve model, the authors analyzed the changing trend of elderly people’s cognitive abilities with age. The influences of community-enriched environments on cognitive abilities were also investigated. Results show that when all the factors are out of consideration except age, for an older person aged 82.5 years, as he/she grows one year older, his/her cognitive abilities will be reduced by 0.139 points, while for one aged 92.5 years, they will be reduced by 0.199 points, which means cognitive abilities decline rapidly as the individuals grow older. The elderly people from communities with enriched environments have higher cognitive levels and slower declining speeds of cognitive abilities than the other elderly people, proving the long-term ability of such environments to facilitate cognitive abilities. An increase in the stimulation of the enriched environment is needed to prevent or slow down the degeneration of cognitive abilities.

## 1. Introduction

Currently, the populations of most countries are aging rapidly, not only of developed countries such as the US and Japan but also of developing ones such as China and Russia. Family size decreases due to a low birthrate, in spite of various birth stimulus policies. Better healthcare driven by socio-economic development and the consistent increase of healthcare input extends life expectancy of older Chinese adults who are suffering from chronic diseases. However, this also leads to an increase in cognitive impairments among older adults. Cognitive functions refer to memory, thinking, reasoning, problem-solving, planning, and processing speed and are also broadly described as aspects of human intelligence [1]. Cognitive impairments refer to one or several of the cognitive functions mentioned above being impaired, thus influencing the memory, learning, and decision-making abilities of individuals in their daily life [2]. Aging is the most significant factor affecting the decline in people’s cognitive functions. With advancing age, elderly people usually experience a decline in these functions. Serious impairments might result in neurodegenerative diseases such as Alzheimer’s disease, not only lowering their living standards but also depriving them of self-care abilities. The obvious increase in demands of treatments and recoveries requires the urgent intervention of families and social resources. In 2010, China estimated 5.69 million people had Alzheimer’s disease and 9.19 million had dementia [3]. By 2050, the World Health Organization predicts that 10 million elderly people in China will have dementia [4].

Generally, elderly people’s cognitive functions decline longitudinally, and such decline accumulates over time. The declining speeds vary among elderly people with different features. Some elderly people have cognitive decline in the early stages of old age, while others maintain good cognitive abilities into old age. At the same time, social development has strengthened the internal heterogeneity of the health of the elderly. Therefore, we need to study the longitudinal declining trend of their cognitive functions (i.e., cognitive trajectories) and explore risk factors causing the decline and interventions.

The limited treatments and medicine effects for neurodegenerative diseases such as Alzheimer’s disease and the increasingly acute problems of adverse reactions and drug tolerance call for the development of new prevention and treatment methods. In 1947, Hebb put forward the concept of “enriched environment” [5], and from then on, this model has been widely used to study the influence of environmental factors on brain functions. In 1978, the enriched environment was first defined as an environment with intervention factors and a complicated combination of nonliving things and social stimuli [6]. It included two categories: socially enriched stimuli and physically enriched stimuli. The former increase the opportunities for interaction among individuals, and the latter provide a complex and changing environment where individuals live by changing the setting or appliances in the environment or by adding different kinds of food and nutrition [7]. The concept of the enriched environment has been widely used in animal experiments. These experiments show that enriched environments can prevent and cure neurodegenerative diseases to a certain degree and increase the effects of other treatments [8,9]. In clinical fields, certain effects have been achieved in recovery with enriched environments, such as the rehabilitation training of paralytics. However, the knowledge of this concept has not been unified because not many people have been tested.

Cognition can be shaped in both neural structures and neurological functions. Under certain conditions, living environments can promote the reshaping and growth of nerves of organisms. Elderly people can compensate for their declining cognitive abilities by optimizing themselves and environmental resources. As a habitation, communities serve as an environment that elderly people have close contact with. Due to the different capacity of community building, the environment of different communities is quite different. An enriched community environment means increased opportunities for interactions, exchanges, and exercises in communities, providing additional stimuli and advancing the growth and development of nerves. However, there is still a lack of systematic analysis on the effects of such an environment in slowing down the decline of cognition functions in the academic circle. The research aims to investigate the roles of such an environment in maintaining elderly people’s cognitive functions to provide beneficial theoretical support to maintain the functions and slow down the declining speed.

## 2. Literature Review

The construction of an enriched environment refers to interventions aimed at promoting activities by creating a stimulating environment. Though much research on the enriched environment theory has been conducted on the neurodegenerative diseases of mammals in labs and apparent effects have been achieved, its applications in practices are quite rare. A few studies on paralytics in rehabilitation wards of hospitals have been carried out. In these studies, a public area for paralytics was constructed, various activities (such as rehabilitation training, group meetings, emotional support from family members, community breakfasts and lunches, and sports) were organized, and equipment (iPads, books, jigsaws, newspapers, games, music, magazines, etc.) was provided. A reference group was also arranged. Patients exposed to an enriched environment were checked for their recovery, and it was found that such an environment facilitates their cognitive functions [10,11,12]. A scholar conducted a psychological counseling intervention with an elderly male patient with dementia for as long as six months. During this period, the scholar made full use of the enriched environment theory and complete support from the patient’s family members, adjusted and improved the patient’s conditions with emotional support (caring for daily life, family dinners, regular exercises, excursions, and constant contact with different parks, shops, and supermarkets) under the enriched environment, and achieved certain effects [13]. From the above research, it was discovered that clinical interventions implemented with this theory generally cover three aspects: activity participation, emotional support, and health care. Practices also prove their role in improving elderly people’s cognitive functions.

Though the enriched environment theory was not mentioned in much other research, all these studies have proved that the above three aspects can improve elderly people’s cognitive functions. Frequent activity participation can advance these functions and slow cognitive aging [14,15,16,17]. Emotional support can protect cognitive functions and slow down the declining speed. Research findings show that the emotional support given by intimate relationships can generate significantly active impacts on cognitive functions [18]. Elderly people who obtain highly frequent emotional support enjoy improved comprehensive cognitive abilities [19]. Other research further finds a close relationship between emotional support and the inhibiting ability of executive function [20]. Advances in health care and health consciousness improve the health of residents [21,22].

In addition, research has shown that elderly people’s cognitive functions are also influenced by many other factors, including demographic factors, socioeconomic factors, lifestyles, self-care abilities, and physical functions. Research proves that aging is associated with a decline of cognitive functions and that age explains to a large degree the differences in the decline over time among different groups of people [23,24,25]. Gender is also an important influencing factor, and females are at a greater risk of cognitive impairment than males [26,27,28]. Adverse factors in socioeconomic aspects, such as low levels of education and poor economic conditions, increase the risk of cognitive impairment [29,30]; place of residence is also a major factor affecting these phenomena. Due to the increased complexity of their working environments and closeness of their interpersonal communications, elderly people in urban areas enjoy better cognitive levels than elderly people in other areas [31]. The lack of activity of daily living (ADL) and instrumental activity of daily living (IADL) is a risk factor influencing these functions [32]. Moreover, hearing disorders mean reduced communication with the external world, increasing the risk of neurodegenerative diseases [33].

The community environment, including the physical and social environment, has been identified and associated with various health outcomes of the elderly [34,35]. Community physical environment refers to the physical resources and/or problems of the community. It consists of roads, outdoor space, transportation, public services, medical and health facilities, and other infrastructure. Social environment refers to the social resources and/or problems of the community. It includes social organizations, sources of social support and opportunities to participate in social leisure activities [36]. Previous studies have shown that communities with less material and social resources are associated with poor cognitive function [37,38,39].

**Figure 1:**
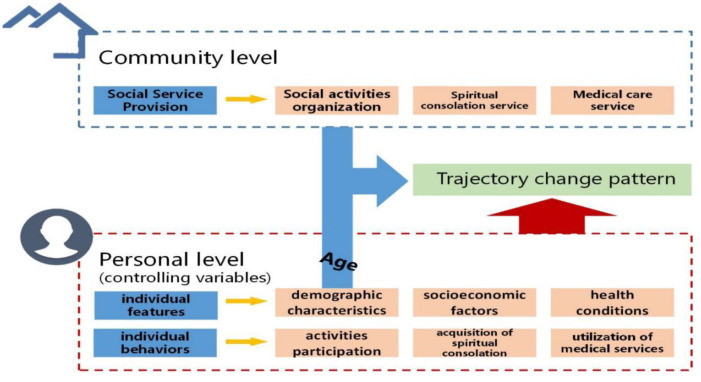
The frame diagram of the theoretical analysis.

In summary, previous research in academic circles has made relatively complete explorations of elderly people’s cognitive functions, mostly from perspectives of social medicine and professional medicine, traced direct and indirect reasons of their cognitive impairments, and discussed the directions and methods of influence of social and demographic factors on cognitive abilities. However, the existing literature about elderly people’s cognitive functions is mostly cross-sectional research, that is, discussing cognitive performance at one particular point in time, or the decline of these abilities is defined as the fractional change between two time points or as cognitive behaviors that decrease below a stipulated threshold. There is a severe lack of longitudinal explorations of these functions based on long-term observation data at many time points. Most previous tracking studies were limited to the linear development of elderly people’s cognitive functions and ignored other possible nonlinear trends. Additionally, existing research mainly focuses on discussing the self-conditions or family environments of elderly people. Systematic and in-depth discussions still need to be made on the influences of the community-enriched environment on cognitive changes with communities serving as a field of activity. Corresponding to the clinical trials, we choose community to organize social activities, community to provide spiritual comfort services, and community medical services to represent the community rich environmental resources. These three aspects are the activities with the most contact and the highest demand for the elderly in the community, which may have a long-term impact on the cognitive trajectory of the elderly. The frame diagram of the theoretical analysis is shown in Figure 1.

## 3. Materials and Methods

### 3.1. Sample

The data used in this research study are from the waves of 2005, 2008, 2011, and 2014 of Chinese Longitudinal Healthy Longevity Survey, which covered 23 provinces and has been conducted for seven rounds in each of 1998, 2000, 2002, 2005, 2008, 2011, and 2014 (See the survey website for details). Due to severe misreporting of the age of elderly people above 105 years, the research focused on those aged 65–104 years. The growth curve model ensured the full use of data from all subjects having experienced at least one cognitive test. Subjects having experienced at least one in four cognitive tests were included in the investigation. In 2005, 2008, 2011, and 2014, 10,057, 3994, 2387, and 1749 effective samples, respectively, were tracked for the survey.

In the baseline survey in 2005, female elderly people accounted for a higher proportion than males, while males did so in the three later surveys. In 2005, 2008, and 2011, the elderly people from rural areas accounted for highest proportions, but the proportion of those from towns exceeded that from rural areas in 2014. Most elderly people lived in ordinary economic conditions, and the smallest fraction were not rich. In 2005, 2008, 2011, and 2014, the elderly people’s average ages were 82.54 years, 80.77 years, 81.14 years, and 82.28 years. Their average number of education years in the four surveys was approximately three, and the average points of the cognitive abilities were approximately 27 (See Table 1).

### 3.2. Measures

#### 3.2.1. Cognitive Ability

The dependent variable is the cognitive ability. According to the Mini-Mental State Examination (MMSE) designed by Folstein, cognitive abilities of the elderly mainly include conditions in direction orientation, calculation, memory, attention, etc. [40]. In the US and Europe, this MMSE is now being widely used and has almost become a standard rating scale. This scale system is also adopted for data in this paper, while the differences are as follows: the ten items of time and direction orientation in MMSE are cut to only five; one instruction item and one sentence-making item are deleted, and one extra item is added by the questionnaire designer.

The revised MMSE consists of 24 questions with a total of 30 points. Among these, there are (1) five questions about direction orientation at one point for each question; (2) three questions about immediate memory (or instantaneous memory) at one point for each question; (3) five questions about attention and calculation abilities at one point for each question; (4) three questions about recent memory at one point for each question; (5) three questions about object confirmation and identification (or object naming) at one point for each question; (6) one restatement about speech for one point and two questions about speech understanding at one point for each question; (7) one question about graph tracing for one point; and (8) another newly added question: name seven things they can eat for a total of seven points at one point for each thing, and seven points for seven or more. The total score is the sum of the scores of each item. The score range is 0–30. The higher the score is, the better the cognitive function is.

#### 3.2.2. Community-Enriched Environment

The independent variables include the community-enriched environment and age (and the quadratic component of age). In this research, the community-enriched environment is defined as communities that organize social activities and provide spiritual consolation services and health care services, and the influences of such an environment on the cognitive trajectories are particularly investigated. The issue of “whether communities organize social and entertaining activities” is discussed for the first aspect. The issue of “whether communities provide spiritual consolation and chatting service” is discussed as the second aspect. Finally, the issues of “whether communities make house calls and give medicines” and “whether communities provide health knowledge” are discussed as the third.

#### 3.2.3. Covariates

Controlling variables include individual features and behaviors. Individual features refer to demographic factors (genders), socioeconomic factors (places of residence, education background, and economic conditions), and health conditions (ADLs, IADLs, and hearing disorders). Individual behaviors include participating in activities, obtaining emotional support, and utilizing medical services. Among these, the first aspect includes individual activities (such as having pets, watching TV, reading books and newspapers, and growing flowers) and organized social activities; the second is decided by whom the elderly people talk to the most frequently, and the last is defined by whether those aged 60 years can seek medical service in a timely manner.

### 3.3. Strategies

Growth curve model was mainly used in this research. Researchers employed a linear growth model and a nonlinear growth model to fit the changing trends of cognitive abilities while ageing. Both models included a random intercept model and a random slope model. Take the linear growth model as an example:

Suppose that older persons were observed several times at T observation time points. $y_{\mathrm{ij}}$ represents the cognitive abilities of the j-th older person at the time point of i, and $t_{\mathrm{ij}}$ is the time when he/she was observed for the i-th time. In this research, $t_{\mathrm{ij}}$ refers to the age of the j-th older person when he/she was observed for the i-th time, i.e., the explanatory variable in Level 1.

In the random intercept model, the cognitive abilities of the elderly are decided by age linearly, which can be expressed as:

$$y_{\mathrm{ij}}=\beta_{0j}+\beta_{1}t_{\mathrm{ij}}+e_{\mathrm{ij}}\;\beta_{0j}=\beta_{0}+u_{0j}$$

In the above equation, $\beta_{0}$, the overall intercept, can be explained as the expected value of y, the cognitive abilities of the elderly at any time point, when $t_{\mathrm{ij}}=0$.

$\beta_{1}$, also known as the growth rate, is the slope of y to the regression line of age. In the random intercept model, it is supposed that the growth rates are the same for all individuals.

$u_{0j}$, the random effect of individual levels, represents the influence of unobserved individual features not changing with age on y and is the difference between y and $\beta_{0}$, the population mean. The variance of $u_{0j}$ is the interindividual differences of y after controlling for the linear influences of age.

In the random intercept model, the variance among the elderly is $\mathrm{var}\left(u_{0j}\right)=\sigma_{u0}^{2}$, which does not change over time.

$e_{\mathrm{ij}}$, the residual of time point level, represents the influence of unobserved individual features changing with time on y; the variance of $e_{\mathrm{ij}}$ is the interindividual differences of y after controlling the linear influences of the time.

The random slope model is obtained by extending the former model and can be expressed as:

$$y_{\mathrm{ij}}=\beta_{0j}+\beta_{1j}t_{\mathrm{ij}}+e_{j}\;\beta_{0j}=\beta_{0}+u_{0j}\;\beta_{1j}=\beta_{1}+u_{1j}$$

The j in the slope parameter is the individual variation in slopes. The growth rate of the j-th older person equals $\beta_{1}$, the average slope in general, plus $u_{1j}$, the stochastic term of the j-th older person.

This research first estimated the random intercept model and the random slope model only with age (and the quadratic component of age) and determined the functional form of cognitive abilities changing over time. Then, researchers took communities organizing social activities, communities providing spiritual consolation services and health care services, and age (and the quadratic component of age) as independent variables and gender, place of residence, education years, etc. as control variables, to analyze their influence on cognitive abilities. To investigate the differences in the declining speeds of cognitive abilities over time of elderly people living in different community environments, the interaction terms of age and community environment and the quadratic component of age and community environment were added to the above models. In the end, the influence of different community environments on the cognitive trajectories and the declining speeds of these abilities in the elderly were compared and analyzed in graphical form.

## 4. Results

### 4.1. The Changing Curve Models of Elderly People’s Cognitive Abilities

Advancing age is the principal factor affecting elderly people’s cognitive abilities. To clarify the changing trend over time, the random intercept model and the random slope model with only age were estimated in this research (See Table 2). For the convenience of explanation, age was centralized depending on the average age in the base survey (approximately 82.5 years). The LR test (Prob ≥ chibar2 = 0.0000) showed that the two models were better than the regression model of a single level. Among these, Model (1), the random intercept model only with age, showed that the overall intercept of elderly people aged 82.5 years (i.e., the average cognitive abilities) was 26.679 points, and the variance of the intercept was 4.631; one year older reduced the cognitive abilities by 0.144 points. The ratio of individual differences to residual variances was approximately 4.631/(4.631 + 9.443) ≈ 32.9%, i.e., the correlation coefficient of two surveys of the same elderly person was 0.329.

Model (2), the random slope model with only age, showed that the overall intercept (i.e., the average cognitive abilities) of elderly people aged 82.5 years was 26.737 points, and the variance of the intercept was 2.655; one year older would reduce the cognitive abilities by 0.143 points, and the variance of the slope would become 0.026. The LR test showed that Model (2) is better than Model (1), with obvious differences between the two (Prob > chi2 = 0.0000).

To check whether there are other functional forms for the declining cognitive abilities, this research added a quadratic component of age based on the former two models (see the latter columns of Table 2). Model (3) was the random slope model of age after its quadratic component was added; Model (4) was the random slope model of age and its quadratic component after the component was added. Different from Models (1) and (2), the quadratic components of age were both obvious at the level of 0.001 in Models (3) and (4), meaning that it was inappropriate to fit the trend of cognitive abilities with linear models. The coefficients of age and its quadratic component were both negative, meaning that the abilities decline faster with advancing age. The decline rate of the abilities can be expressed as:

$$\frac{{d\;\mathrm{cog}}_{\mathrm{ij}}}{d\;\mathrm{age}82.5_{\mathrm{ij}}}=-0.139-2\left(0.003\right)\mathrm{age}82.5_{\mathrm{ij}}$$

For example, for an older person aged 82.5 years, as he/she grows one year older, his/her cognitive abilities will be reduced by 0.139 points, while for one aged 92.5 years, they will be reduced by 0.199 points. The LR test discovered that there was no obvious difference between Model (3) and Model (4). Therefore, Model (3) is taken as the reference model to be analyzed in this research (i.e., cognitive abilities are a quadratic function of age).

### 4.2. Multilevel Regression Model of Changes in Cognitive Abilities

To further explore and analyze the influence factors of changes in cognitive abilities on the basis of Model (3), i.e., the random slope model only with age and its quadratic component, communities organizing social activities and providing the spiritual consolation and medical care services were taken as independent variables, and gender, place of residence, ADLs, IADLs, education years, socioeconomic status, participation in social activities, and obtaining spiritual consolation and medical care services were taken as control variables in Model (5) to analyze the influence of these variables on cognitive abilities. (see Table 3)

To investigate the differences in the declining speeds of cognitive abilities over time of elderly people living in different community environments, the interaction terms of age and community environment and the quadratic component of age and community environment were added to the above models. Based on Model (5), the interaction terms of age and social activities organized by communities and of the quadratic component of age and these activities were added in Model (6). Based on Model (5), the interaction terms of age and the spiritual consolation service provided by communities and of the quadratic component of age and this service were added in Model (7). Depending on Model (5), the interaction terms of age and the medical care service provided by communities and of the quadratic component of age and this service were added in Model (8).

Research findings showed that cognitive abilities decline with advancing age. The intercept term was 27 points, which was the score of the elderly with an average age of 82.5 in the baseline survey. Because the coefficients of age and its quadratic component were both negative in Models (5), (6), (7), and (8), the cognitive abilities declined with increasing speed as elderly people aged.

Model (5) showed that the cognitive abilities of those whose community organizes social activities were increased by 0.244 points. The cognitive abilities of those whose community provides spiritual consolation services were increased by 0.401 points. The cognitive abilities of those whose community provides medical care services were increased by 0.165 points.

In Model (6), effects of social activities organized by communities were not apparent, showing that whether communities do so or not will generate little influence on cognitive abilities of those with an average age of 82.5 at baseline. The coefficient of age and its quadratic component showed that the cognitive abilities of those whose communities do not organize activities decreases in a curved pattern; the obvious coefficient of the interaction term of age and communities doing so demonstrated differences in age patterns of cognitive abilities between the two groups of those in communities organizing activities and those in communities that do not.

In Model (7), the coefficient of communities providing spiritual consolation services was obvious, meaning that communities doing so will exert positive influences on the cognitive abilities of those with an average age of 82.5 at baseline. The coefficient of age and its quadratic component showed that the cognitive abilities of those in communities providing the service decreases in a curved pattern; the obvious coefficient of the interaction term of age and communities doing so demonstrated differences in age patterns of cognitive abilities between the two groups of those in communities providing the service and those in communities that do not.

In Model (8), the coefficient of communities providing medical care services was obvious, meaning that communities doing so will have positive influences on the cognitive abilities of those with an average age of 82.5 at baseline. The coefficient of age and its quadratic component showed that the cognitive abilities of those in communities providing the service decreased in a curved pattern; the obvious coefficient of the interaction term of age and communities doing so demonstrated differences in age patterns of cognitive abilities between the two groups of those in communities providing the service and those in communities that do not.

### 4.3. The Heterogeneity of Changes in Cognitive Abilities

To intuitively reflect on the cognitive abilities of elderly people living in different community environments and differences in their declining speeds, we showed the interaction results of the environments and age and its quadratic component (Models 6, 7, and 8) in the form of graphs.

**Figure 2:**
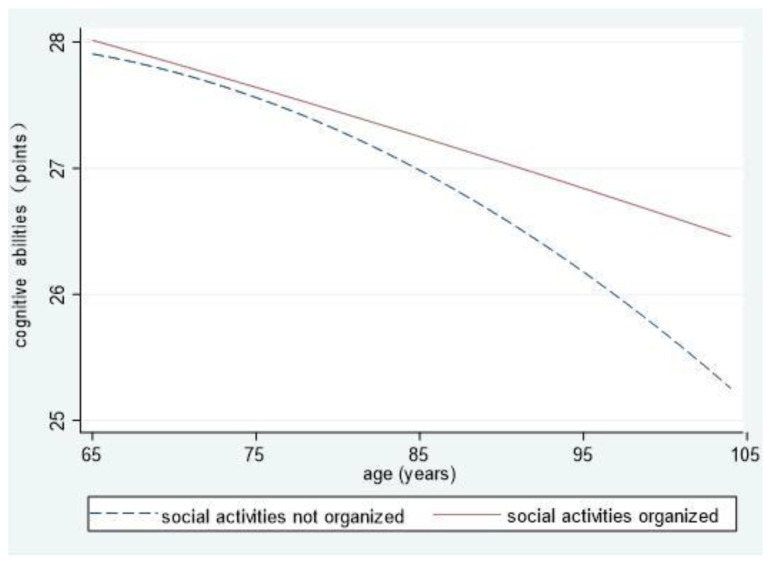
The influences of communities organizing social activities on cognitive abilities.

From Figure 2, we can see that the cognitive abilities of elderly people in communities organizing social activities are always higher than the cognitive abilities of those in communities not doing so, and the abilities of the two groups decline at different speeds. Concretely speaking, the cognitive abilities of the former group decline in a linear and stable manner, and those of the latter decline in a curved and rapid manner. The decline accelerates after the oldest-old age period is entered, resulting in increased differences with advancing age.

**Figure 3:**
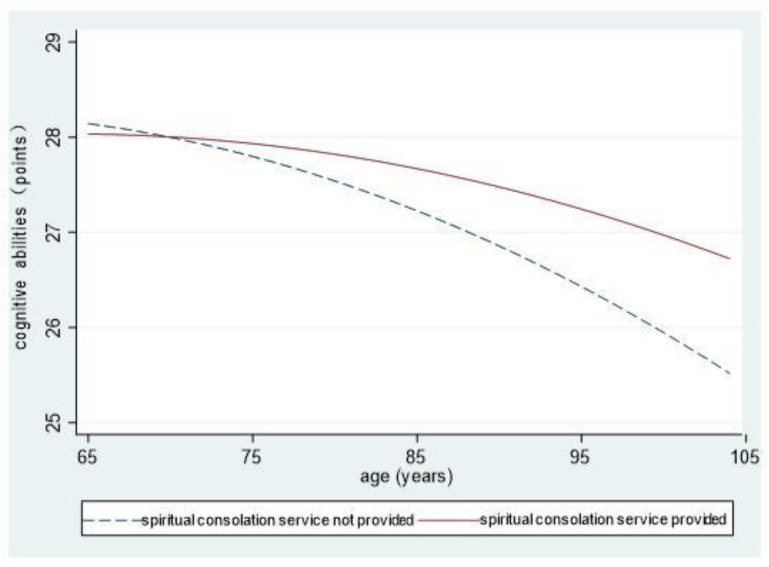
The influences of communities providing spiritual consolation services on cognitive abilities.

Figure 3 shows no obvious differences in cognitive abilities between elderly people in communities providing these services and those in communities not doing so after they enter the old age period. Later, cognitive abilities of the latter group decline at increased speeds, while such abilities of the former decline in a gentle and curved manner. In addition, cognitive abilities of the former are better than those of the latter, and the differences increase with advancing age.

**Figure 4:**
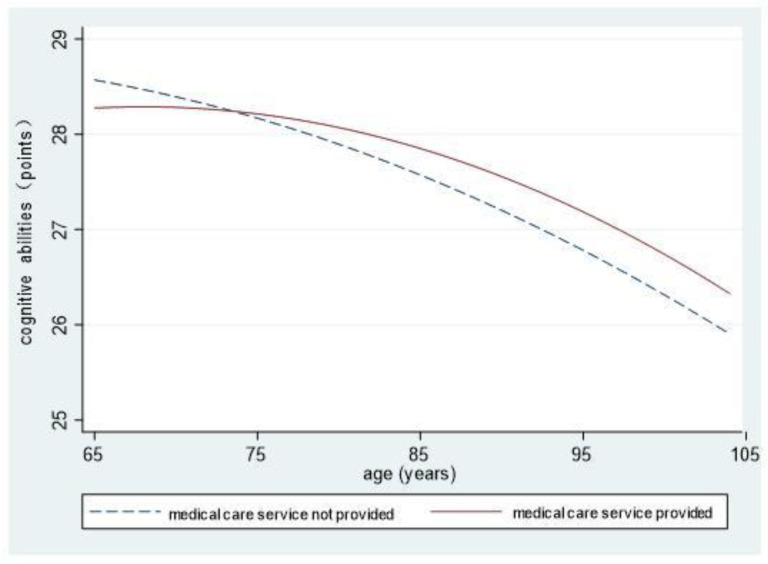
The influences of communities providing medical care services on cognitive abilities.

Figure 4 shows that the cognitive abilities of elderly people in communities providing medical care services are higher than the cognitive abilities of those in communities not doing so for most of the old age period. The abilities of the two groups both decline in a curved manner, and those of the latter decline faster. Differences increase with advancing age.

## 5. Discussion

In this research, the growth curve model is used to explore the nonlinear change model of elderly people’s cognitive trajectories, increase the reliability of assessments of long-term cognitive changes, and mainly discuss the influences of community-enriched environments on cognitive trajectories. Research results are of great theoretical and practical significance for maintaining the cognitive abilities of elderly people and slowing down their decline.

### 5.1. The Changing Trend of Elderly People’s Cognitive Functions

Research findings show that the cognitive abilities of elderly people decline in a nonlinear and accelerated manner with advancing age. Generally, slowly declining cognitive function means slight cognitive disorders. However, after this stage, these abilities decline in an accelerated manner, and many elderly people will even suffer from senile dementia. Knowledge of this fact helps us have a clear understanding of the cognitive trajectories of elderly people and grasp the best intervention time points. Before the rapid decline of cognitive functions, timely intervention and enhancing the stimulation of enriched environments can prevent or slow down further decline.

### 5.2. Community-Enriched Environments and Cognitive Trajectories

Obvious impacts will be generated on elderly people’s cognitive trajectories—both in levels and in decline speeds—if their communities organize social activities and provide spiritual consolation services and medical care services. They will enjoy increased cognitive levels and a slowed decline in abilities.

Research in neuroscience has shown that cognition can be shaped in both neural structures and neurological functions. Under certain conditions, living environments can facilitate the reshaping and growth of nerves of organisms, i.e., cognition changes with surrounding environments. On the one hand, an enriched community environment means additional opportunities for interactions, exchanges, and exercises in communities, providing additional stimuli and advancing the growth and development of nerves. From this perspective, the community-enriched environment directly facilitates the promotion of elderly people’s cognitive abilities. On the other hand, according to the theory of cognitive reserves, individual self-adaption constantly compensates for the increasing brain injuries with the neural network, thus buffering the crippling effects of brain injuries in clinical manifestations. Self-adaption enables individuals to stand up to brain injuries and ensures optimized clinical manifestations or behaviors [41]. Previous research found that participation in activities and emotional support can help accumulate cognitive reserves and maintain or improve elderly people’s cognitive functions [42]. Our research agrees with this idea. We also investigated communities as a cognitive reserve environment and further verified the protective effects of communities organizing social activities and providing two services on cognitive levels and the declining cognitive abilities. Unlike previous studies that showed that the emotional support given by intimate relationships can generate significantly active impacts on cognitive functions, our study found that community can play a similar role. Undoubtedly, it has important implications for a society with accelerated population flow, serious empty-nested phenomenon, and frequent neglect of the elderly. Being different from direct promotion, this mechanism of action is more likely to be indirect protection. In addition, previous research did not reach an agreement on the relationship between cognitive reserves and advancing age [43,44]. Our research proves that the effects of community-enriched environments as cognitive reserves increase with advancing age. For example, communities organizing social activities slow down the decline of cognitive abilities, and this effect becomes more obvious as elderly people grow older, which might be due to the compensation of cognitive reserves.

Our study has validated the role of community health care services in mitigating cognitive decline of elderly people. But it is interesting to note that this effect was not found in another study on the elderly in urban China [45]. It is probably because comparing to rural areas, there are more big hospitals in cities and towns that enhanced the accessibility of healthcare service for the elderly in urban areas. Therefore, community medical and healthcare has greater significance to the rural elderly.

Communities, important to the lives of elderly people, are places where they can exchange information, learn about society, and obtain resources. They are able to compensate for the limited resources from family lives, provide more stimuli, and generate imperceptible influences on the cognitive functions of the elderly people. On the one hand, communities where elderly people live should actively organize social activities and make more efforts to provide spiritual consolation services. Communities should also enhance the construction of places and facilities of exercises and recreation to create more opportunities for spiritual and cultural communication and entertainment in the neighborhood. Communities should perfect barrier-free facilities to make it easier for elderly people to get around. On the other hand, timely seeking of medical services is a difficult and key point in elder care within families. It is suggested to build and improve the information platform of elderly care service in the community to meet the elderly’s needs of medical care, rehabilitation, and nursing service, to carry out the medical concept of relying mainly on prevention and integrating prevention with cure in order to improve the health quality of the residents comprehensively.

### 5.3. Advantages, Shortages, and Prospects

The growth curve model was employed to analyze the nonlinear changing patterns of elderly people’s cognitive trajectories. It was found that their cognitive abilities decline with advancing age in a nonlinear pattern and that the decline accelerates as they grow older. Previous research mostly regarded the changing trend as linear and ignored the nonlinear pattern. In addition, this research also proved the long-term facilitation effects of community-enriched environments on cognitive functions. Previous research did not reach an agreement on the relationship between cognitive reserves and advancing age, while our research proves that the effects of such an environment grow as cognitive reserves and develops the theory of cognitive reserve to a certain extent.

This research has certain limitations. The samples are mostly the oldest-old, so the results may be influenced by the choice effect of death. Elderly people who survive to old age are generally in better health, while those who don’t survive to old age have relatively poor health—that is, lower cognitive scores. Therefore, the proportion of oldest-old in our sample may result in increased average points of cognitive abilities. However, to a certain degree, this research reveals the changing trend of elderly people’s cognitive trajectories and important effects of community-enriched environment, which is of great significance to future research.

In summary, the community-enriched environment only requires the purchase of some facilities and fewer professionals. It is a convenient, effective, and cost-efficient way to improve elderly people’s cognitive abilities within a budget and is worth being perfected and promoted. It is worth noting that indexes of the enriched environment are limited to communities organizing social activities and providing spiritual support and medical care services due to the limitation of these data. The connotation of an enriched environment goes far beyond this. In addition to what was mentioned above, we need to consider what elements can be constructed to develop such an environment and how to make the best of its effects. For instance, an environment with complete smart elder care facilities and barrier-free facilities is a kind of enriched environment. However, the same environment is enriched to someone with a positive attitude, yet an impoverished one for someone with a negative attitude. Based on developing the best enriched environment, we must mobilize the subjective initiative of elderly people, and only through interactions between people and the environment can its effects be fully played out and understood.

## Figures and Tables

**Table 1:** Basic conditions of elderly people (n/%).

Features		2005		2008		2011		2014	
		n	%	n	%	n	%	n	%
Gender	Male	4940	49.12	2091	52.35	1239	51.91	899	51.40
	Female	5117	50.88	1903	47.65	1148	48.09	850	48.60
Place of residence	Urban areas	2504	24.90	915	22.91	548	22.96	378	21.61
	Towns	2027	20.16	851	21.31	836	35.02	696	39.79
	Rural areas	5526	54.95	2228	55.78	1003	42.02	675	38.69
Economic conditions	Rich	1789	17.79	555	13.90	492	20.61	348	19.90
	Ordinary	6815	67.76	2807	70.28	1517	63.55	1228	70.21
	Not rich	1453	14.45	632	15.82	378	15.84	173	9.89
Average number									
Age	(years)	82.54		80.77		81.14		82.28	
Education	(years)	2.64		2.99		3.18		3.07	
Cognition	(points)	26.64		26.93		27.20		27.30	

**Table 2:** The random effect of changes in elderly people’s cognitive abilities.

Variables	(1) Random Intercept	(2) Random Slope	(3) Random Slope	(4) Random Slope
	Regression Coefficient	Regression Coefficient	Regression Coefficient	Regression Coefficient
Age	−0.144 ***	−0.143 ***	−0.139 ***	−0.138 ***
Age^^2^			−0.003 ***	−0.003 ***
Intercept	26.679 ***	26.737 ***	26.945 ***	26.945 ***
Variance (age)		0.026	0.026	0.000
Variance (age^^2^)				0.000
Variance (intercept)	4.631	2.655	2.654	3.086
Variance (residual error)	9.443	8.846	8.819	8.999
Sample size	18,187	18,187	18,187	18,187
−2LL	98,602	98,240	98,166	98,082

Note: *** *p* < 0.001.

**Table 3:** Multilevel regression model of changes in cognitive abilities.

	(5) No Interactions	(6) Communities Organize Social Activities-Age Interactions	(7) Community Spiritual Consolation Service-Age Interactions	(8) Community Health Care Service-Age Interactions
Variables	Regression Coefficient	Regression Coefficient	Regression Coefficient	Regression Coefficient
Age	−0.060 ***	−0.063 ***	−0.063 ***	−0.064 ***
Age^^2^	−0.001 ***	−0.001 ***	−0.001 ***	−0.001 **
Female	−0.650 ***	−0.648 ***	−0.651 ***	−0.647 ***
No hearing disorders	1.699 ***	1.695 ***	1.700 ***	1.700 ***
ADLs	−0.070 **	−0.073**	−0.072 **	−0.071 **
IADLs	−0.185 ***	−0.185 ***	−0.185 ***	−0.185 ***
Education years	0.126 ***	0.126 ***	0.126 ***	0.126 ***
Economic conditions				
Ordinary	−0.224 **	−0.221 **	−0.223 **	−0.220 **
Not rich	−0.501 ***	−0.501 ***	−0.502 ***	−0.498 ***
Place of residence				
Town	−0.288 ***	−0.289 ***	−0.287 ***	−0.289 ***
Village	−0.425 ***	−0.426 ***	−0.422 ***	−0.423 ***
Timely seeking of medical service in serious illness	0.771 ***	0.767 ***	0.7682 ***	0.766 ***
Whom to chat with				
Others	−0.335 ***	−0.343 ***	−0.342 ***	−0.341 ***
No one	−1.146 ***	−1.149 ***	−1.144 ***	−1.146 ***
Participation in social activities	0.263 ***	0.271 ***	0.267 ***	0.272 ***
Engage in recreational activities individually	1.151 ***	1.137 ***	1.145 ***	1.141 ***
Community organizes social activities	0.244 **	0.197	0.249 **	0.244 **
Community provides the spiritual consolation service	0.401 ***	0.384 ***	0.357 **	0.387 ***
Community provides the medical care service	0.165 **	0.163 **	0.164 **	0.226 **
Age * Community organizes social activities		0.024 **		
Age^^2^ * Community organizes social activities		0.001		
Age * Community provides the spiritual consolation service			0.032 **	
Age^^2^ * Community provides the spiritual consolation service			0.000	
Age * Community provides the medical care service				0.021 **
Age^^2^ * Community provides the medical care service				−0.001
Intercept	27.113	27.154	27.144	27.117
Variance (age)	0.019	0.019	0.019	0.019
Variance (intercept)	1.258	1.253	1.258	1.253
Variance (residual error)	8.354	8.356	8.350	8.356
Sample size	18,187	18,187	18,187	18,187
−2LL	95,138	95,128	95,128	95,130

Note: *** *p* < 0.001, ** *p* < 0.01, * *p* < 0.05.

## Data Availability

Data can be found from the Chinese Longitudinal Health Longevity Survey (CLHLS).
